# Aging Impairs the Cellular Interplay between Myeloid Cells and Mesenchymal Cells during Skin Healing in Mice

**DOI:** 10.14336/AD.2021.1008

**Published:** 2022-04-01

**Authors:** Saeid Amini-Nik, Abdikarim Abdullahi, Roohi Vinaik, Ren Jie Robert Yao, Nancy Yu, Andrea Datu, Cassandra Belo, Marc G Jeschke

**Affiliations:** ^1^Institute of Medical Science, University of Toronto, Toronto, Canada.; ^2^Sunnybrook Research Institute, Toronto, Canada.; ^3^Laboratory Medicine and Pathobiology, University of Toronto, Toronto, Canada.; ^4^Department of Surgery, Division of Plastic Surgery, University of Toronto, Toronto, Canada.; ^5^Department of Immunology, University of Toronto, Toronto, Canada.; ^6^Ross-Tilley Burn Centre, Sunnybrook Health Sciences Centre, Toronto, Canada

**Keywords:** elderly, wound healing, macrophages, mesenchymal stem cells, burns, thermal injury

## Abstract

Impaired wound healing is a major issue in the elderly population and is associated with substantial health and economic burden, which is exponentially increasing with the growing aging population. While the underlying pathobiology of disturbed skin healing by aging is linked to several genetic and epigenetic factors, little is known about the cell-cell interaction during the wound healing process in aged individuals, particularly the mesenchymal stem cell (MSCs)-macrophages axis. In this study, by using a thermal injury animal model in which we compared the wound healing process of adult and young mice, we found that the insufficient pool of MSCs in adult animals are deficient in migrating to the wound bed and instead are restricted to the wound edge. We identified a deficiency of a CD90-positive MSC subpopulation in the wounds of adult animals, which is positively correlated with the number of F4/80+ macrophages. *In vitro*, we found that CD90+ cells preferentially adhere to the myeloid cells forming doublet cells. Thus, our findings highlight that in adult mice subjected to a thermal injury, impaired wound healing is likely mediated by a disturbed cellular interplay between myeloid cells and mesenchymal cells.

Chronic, non-healing wounds are a substantial challenge for health care system. Health professionals are currently using multiple modalities to heal wounds with unpredictable degrees of success, more often met with failure than success. Inadequate wound closure is a major burden in health care as we face an aging population who are more prone to wound healing deficiencies leading to poor outcomes. This is particularly a concern in burn patients in whom delayed healing increases risk of infection, sepsis, and mortality.

A severe burn is a devastating injury associated with significant morbidity and mortality [1-3]. The prevalence of burns in North America is greater than two million each year [4]. Furthermore, the World Health Organization (WHO) reported an estimated 330,000 deaths per year worldwide related to thermal injury [5]. Clinical and therapeutic advancements in burn care such as early excision and grafting, adequate nutrition, and implementation of critical care bundles, have markedly decreased mortality rates and improved outcomes in pediatric and adult burn patients over the last three to four decades [6].

However, this is not the case in elderly burn patients (65 years of age or greater). Despite current advances, LD50 burn size in elderly patients has remained unchanged during the past three decades, and age-associated morbidities are still unacceptably high. The elderly are a rapidly growing population that are more susceptible to burns, attributable to mental alterations, decreased sensations, and other factors [7, 8]. After injury, impaired skin healing in this population remains a significant issue, with three percent of the population suffering from disordered wound repair [9-11]. It is believed that pre-existing medical conditions [12], failure of the immune system to fight of post-burn infections along with altered inflammatory and immune responses [13], and decreased metabolic resources and capacity [14] are some of the contributing factors in deficient skin healing of the elderly.

We recently conducted a large cohort trial in elderly burn patients that showed a significantly longer hospital stay in the elderly when compared with adults, mostly due to insufficient wound healing and increased complications rates [15, 16]. Impaired wound healing observed in elderly burn patients was associated with 1) reduced pool of Stro-1 positive mesenchymal progenitor cells in their skin 2) a deficient migration of mesenchymal stem cells (MSCs) *in vitro* and 3) a deficient activation of essential signaling pathways (e.g. Wnt/β-catenin) during skin healing [17]. Furthermore, elderly patients demonstrated a failure to initiate the systemic inflammatory cascade, an essential step for optimal skin healing.

Physiological wound healing is divided into the sequential yet overlapping stages of hemostasis, inflammation, proliferation, and remodeling [18, 19]. Skin wound healing is a highly orchestrated physiological process that involves the interplay of keratinocytes, mesenchymal-like fibroblasts, immune cells, and endothelial cells, amongst others [9, 20]. The inflammatory phase has in particular has a determining role, and studies showed that macrophages are essential in shifting the inflammatory to the proliferative phase and are crucial for effective healing [10, 21-23].

Here, we investigated wound healing deficiencies in elderly patients using a rodent burn model. In particular, we assessed the role of myeloid lineage cells and delineate the interplay between myeloid cells and CD90+ mesenchymal cells during skin healing. We aimed to determine if impairments in this cell-cell interplay could partly attribute to the poor healing outcomes in adult versus young mice. Potentially, these findings can be extrapolated to humans and can provide a foundation for clinical investigation on impaired healing in elderly versus young patients.

## MATERIALS AND METHODS

### Mouse Model Thermal Injury

Male C57BL/mice (Jackson Laboratory) were housed at ambient temperature for two weeks and cared in accordance with the Guide for the Care and Use of Laboratory Animals. We used male mice in this study to take into account potential confounders such as variations in hormone physiology. Young mice (8-12 weeks) and aged “adult” mice (52-54 weeks) were used in this study. All procedures performed in this study were approved by the Sunnybrook Research Institute Animal Care Committee. Pre-burned mice were treated with buprenorphine at a concentration of 0.03 mg/mL, diluted to 1:100, and each mouse was injected with 50 μL [24,25]. Unless specified otherwise, full-thickness (20% TBSA) was used to burn the mice by placing animals on their back in a template constructed of a plastic flame resistant with the window exposing a predetermined surface area of skin^26^. We used two sizes of the templates in order to reach 20% TBSA (larger window for aged animals). The animals are then observed frequently for signs of pain or discomfort and treated with buprenorphine or other painkillers as needed. Burned mice were subsequently housed individually in sterile cages and sacrificed.

### Bone Marrow and BD-MSC isolation

Young and adult mouse were euthanized using cervical dislocation. The back legs were removed and dissected away from the muscle around the bones until clean enough to see either end of the leg bones. Using a clean set of scissors and forceps, an incision was made just below the hip joint and above the knee. The bone was held above the tube and a needle was inserted into the bone opening to flush the bone marrow into the tube using 5 mL of 1 X PBS. The bottom part of the leg was cut just below the knee and just above the ankle; 5 mL of 1 X PBS was used to flush the bone marrow into the tube. Large pieces were broken down with a syringe by drawing the sample up and down many times. Then the bone marrow was filtered through 100 μm filters into a new tube and rinsed with extra PBS.

Samples were centrifuged at 1000 rpm for 5 min, and the supernatant was removed. Pellet was treated with 1 ml RBC lysis buffer for 5 min at 37 degrees C. After incubation, we added PBS, centrifuged down 1000rpm for 5 min and remove the supernatant by suction. Pellet was resuspended in MesenCult™ Proliferation Kit with MesenPure™ (Mouse) (Stem Cell Technologies, Vancouver, Canada) to differentiate into MSC’s in 75 cm^2^ flasks.

For macrophage isolation in young and adult mice, the same treatment was done as BD-MSS isolation. However, the pellet was resuspended in RPMI medium: (Wisent Quebec, Canada) supplemented with 10% FBS (Wisent Quebec, Canada), 10% L929 conditioned medium, and 1% antibiotic/antimycotic (Wisent Quebec, Canada). L929 conditioned media was prepared by expanding L929 cells in culture EMEM (Wisent Quebec, Canada), 10% horse serum (Wisent, Quebec, Canada), 1% Ab/Am in the desired flask. This was left for one week without changing medium and filtered through 0.45 μm filter. 50 mL aliquots were and stored at -80 until needed for RPMI media.

### Flow Cytometry

For young and adult murine BD-MSCs, flow cytometry was performed using Negative markers: CD34, CD45, and positive markers: CD73, CD105, and CD90. Live cells were selected and the CD34-/CD45- population was further probed for CD73+/CD105+/90+. Antibodies used were CD34 FITC (Invitrogen), CD45 PerCP/Cy5.5 (Biolegend), CD73 PE (eBioscience) and CD105 APC (eBioscience) and CD 90 APC/Cy7 (Biolegend). Flow was run on LSRII and analyzed using FlowJo.

### Doublet Experiment

MSCs and EYFP+ macrophages were allowed to interact for 6 hours and washed with PBS. Using FACS sorting, CD90+ and CD90- cells were isolated, and doublet cells within CD90+ or CD90- cell populations were gated. In addition, EYFP+ macrophage populations within the CD90+/- doublet cells were also gated for further analysis.

Bone marrow from a young mouse (8 weeks) was cultured in a 10 cm plate with MesenCult media (StemCell Technologies) according to protocol. On day 3, bone marrow from a Lyzs-Cre-ROSA-EYFP mouse was cultured with L929 media for seven days using established protocol. On day 10, 3 million EYFP+ macrophages were added per each of the 10cm plates on top of the MSCs of the same animal. After 6 hours, the unbound cells were washed away with PBS, and the cells were stained with CD90 antibody for FCM.

### Immunohistochemistry & determination of wound width

Paraffin-embedded skin tissue slides were deparaffinized by heating for 30 min at 60°. Slides were then placed in citrosol (2×), 100% Ethanol (2×), 95% ethanol, 70% ethanol for 3 min each, followed by water. Antigen retrieval was then performed using antigen decloaker (1×; Biocare Medical, Concord, CA, USA), which was added to the slides in a preheated decloaking chamber for 4 min at 110°C. For BrdU staining, samples were denatured with 1.5 N HCl for 30 min at 37°C and neutralized with 0.1 M borate buffered twice for 5 min. Samples were blocked with 3% H_2_O_2_ for 10 min and then washed with washing buffer (0.05 M Tris-HCl, 0.15 M NaCl, 0.05% Tween 20 in deionized water). The primary antibody (mouse monoclonal F480, 1:200; AbD Serotec (Biolegend), Mississauga, ON, CAN) was diluted in PBS and incubated at room temperature for 1 h. For CD90, analysis, a different section was probed with CD90 (rat monoclonal, CD90 anti-mouse, 1:200; Abcam, Cambridge, UK) primary antibody diluted in PBS was incubated at room temperature for 1 h. Sections were also treated with Sca1, (rat monoclonal, Sca1 anti-mouse, 1:200 Abcam, Cambridge, UK;). Slides were then incubated for 15 min with MACH3 mouse probe (Biocare Medical), followed by MACH3 mouse horseradish peroxidase polymer, with washes in between. The betazoid diaminobenzidine chromogen kit (Biocare Medical) was mixed and added for 5 min or until brown stain was noticeable. The reaction was terminated with running water. Nuclear staining was carried out with hematoxylin for 30 s, followed by differentiation with three dips in 1.5% acid alcohol and bluing in 0.1% sodium bicarbonate for 10s. Sections were dehydrated through 95% and absolute ethanol to citrosol and mounted with SHUR/Mount. Images were acquired using a Zeiss Axiovert 200 light microscope at 10× magnification to image the whole section followed by 40× magnification to further focus on the wound margins and the wound center. The 40× magnification images were quantified by positive cells using ImageJ software, and then normalized to the number of total cells in the 40× field. Wound width was calculated for young and adult mice (n=5-7) by measuring from the edges of the wound margin (intact skin/healing zone junction). Only areas with granulation tissue were included in the final assessment of wound width.

### Mice irradiation and macrophage treatment

Young mice were exposed to a 2.6-Gy whole-body irradiation using a ^137^Cs irradiator and were wounded 24 hours after irradiation using 4-millimeter punch biopsy. Cultured macrophages (10^5^ cells) from donor mice (same age) were mixed with 50 μl of Matrigel (BD Biosciences) and applied to wounds of recipient mice immediately after injury.

### Statistical analysis

All *in vitro* experiments were performed three times. The comparative statistical analysis was carried out by using the Student’s *t*-test. Correlation between various variables was done using Pearson correlation equation for non-normal variables. Significance levels were set at *P<0.05 and **P<0.01.

## RESULTS

### Deficient skin healing of adult mice

**Figure 1.**
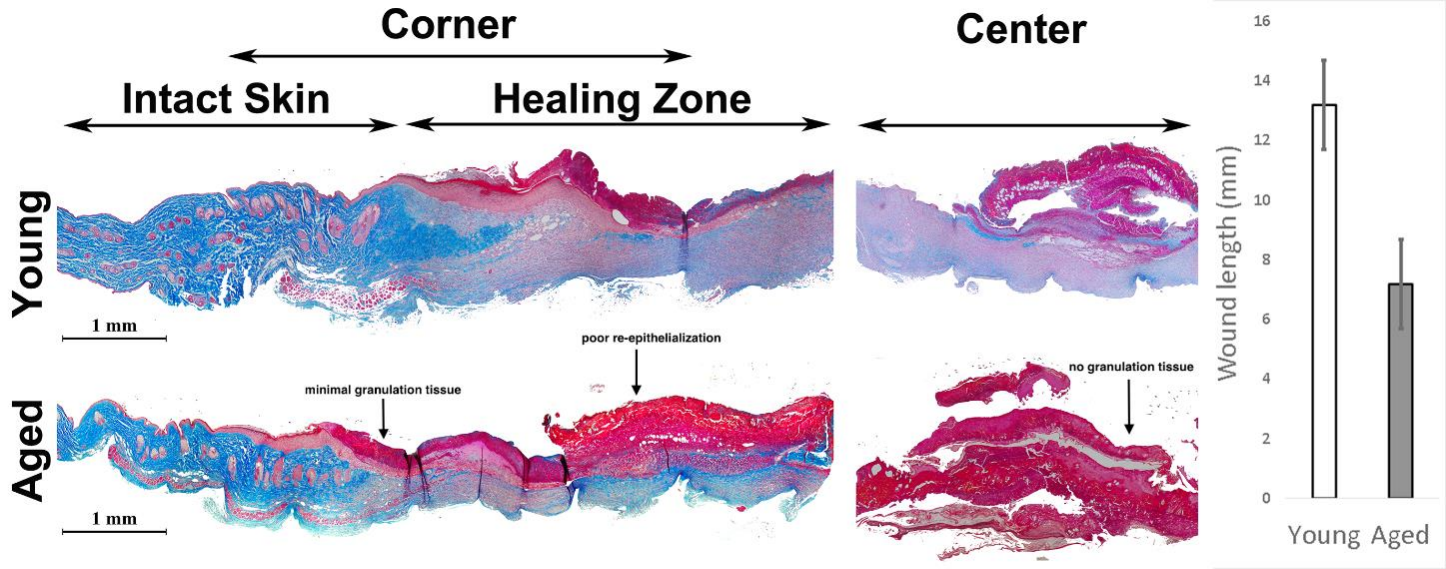
Adult mice show deficient skin healing post thermal injury. Trichrome staining of the edge and the center of the burned area shows superior granulation tissue and healing in the skin of young mice in comparison with adult mice (right). Note the complete lack of granulation tissue, particularly in the center of the damage zone of the adult animal (n=8). Decreased wound length (mm) in adult relative to young mice (left) (n=5-7). (*P< 0.001, error bars are showing 95% confidence intervals). Areas of impaired re-epithelialization and poor granulation are indicated in adult (aged) tissue and marked with an arrow.

To characterize burn wound healing mice, we subjected adult male animals (52-54 weeks old) to 20% total body surface burn injury (Supplementary Fig. 1) and compared outcomes with young male animals (8-10 weeks old). Upon histological examination, we identified deficient skin healing in adult mice two weeks post-burn. In adult animals, minimal granulation tissue formed at the junction of intact skin and active healing area, and the center of the injury showed almost no granulation tissue (Fig. 1). In addition, the extent of re-epithelialized skin in the damaged zone was lower in adult mice. After identifying that adult mice exhibit impaired healing, we subsequently investigated if altered mesenchymal stem cell (MSC) recruitment could account for these findings.

### Deficient skin healing in adult mice is associated with deficient recruitment of MSCs into the wound bed

**Figure 2.**
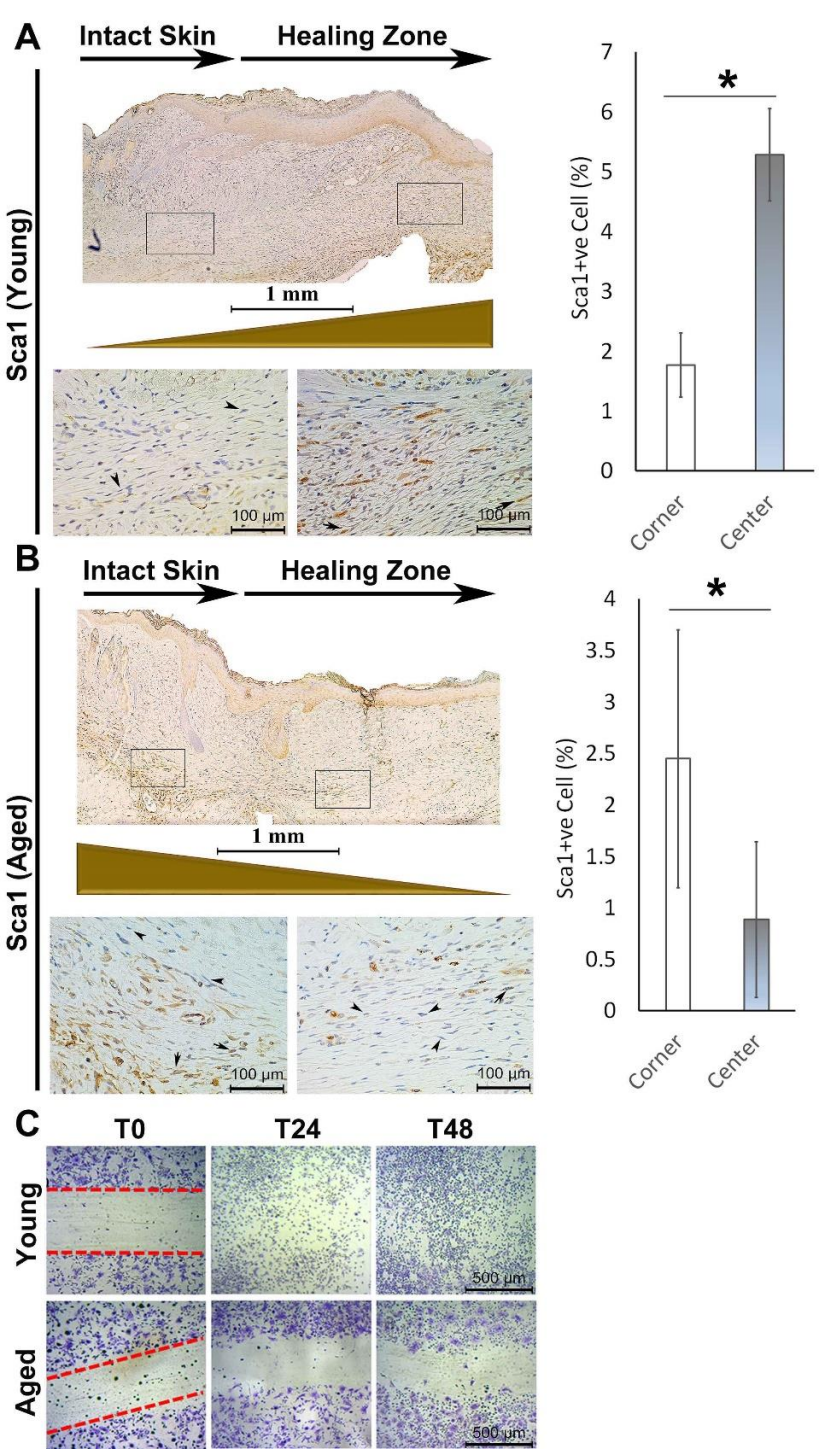
Sca1+ mesenchymal stem cells are restricted to the junction of intact skin in injured adult mice and are enriched in the active healing area in young mice. (A) In young animals, the number of Sca1+ mesenchymal cells is significantly less at the junction of intact skin in comparison with the active healing area toward the center of the wound two weeks post-injury (B) In the adult animal, the number of Sca1+ mesenchymal cells is significantly higher at the junction of intact skin (left call out box) in comparison with the active healing area (right call out box) toward the center of the wound (n=5). (C) *In vitro* scratch assay of BM-MSCs isolated from young vs. adult animals show migration deficiency in the adult group (*P < 0.05, error bars are showing 95% confidence intervals). Arrows indicate Sca1+ cells present in murine dermis.

In humans, we previously reported that mesenchymal cells from the skin of elderly patients are deficient in migration *in vitro*[16]. To explore the spatial distribution of mesenchymal cells in the wound beds of adult and young animals, we stained the wounds with Sca-1 (Stem cell antigen-1). Sca-1 positive MSCs were enriched toward the center of the healing area in the young (Fig. 2A). Unlike young animals, adult mice showed the opposite pattern - a minimal number of Sca-1+ cells at the center of the active healing zone and a greater number of Sca-1+ cells at the junction of intact and healing skin margin. This suggests that in adult mice, recruited MSCs are restricted to the margin of the wound, most probably due to deficient migration ability (Fig. 2B).

### Bone marrow-derived MSCs from the adult animal are deficient in migration in vitro

To further characterize the migration efficiency of mesenchymal stem cells (MSCs) in adult mice, we harvested bone marrow and maintained them in MesenCult™ media according to the manufacturer’s protocol. Once differentiated, we performed a scratch assay on bone marrow-derived MSCs (BM-MSCs) of adult and young animals to assess migration of MSCs. Relative to MSCs from young animals, MSCs of adult animals showed significantly impaired migration *in vitro* (Fig. 2C). Furthermore, we isolated RNA from BM-MSCs of adult and young animals and subjected them to Cell Motility PCR Array (PAMM-128Z). In particular, we demonstrated a 11.9-fold downregulation in DPP4, which is involved in cell adhesion, migration, and apoptosis - processes that are critical for wound healing^27^. BM-MSCs from adult mice also demonstrate a 6.11-fold decrease in EGF that functions via EGFR/ERK1/2 signalling and a 2.53-fold decreased in Fap, impairing cell migration compared to young mice^28,29^. Adult mice also have a 2.99-fold decrease in Cav-1, and cells deficient in Cav-1 lose normal polarity and exhibit impaired wound healing, coupled with decreased Rho expression as illustrated here (4.16-fold decrease relative to young mice)^30^. Taken together, BM-MSCs of the adult animals exhibit a downregulation in several essential genes involved in cell motility, providing a rationale for the impaired healing seen in adult relative to young mice (Supplemental Table 1).

### Reduced F4/80+ macrophages and CD90+ mesenchymal cells in the bone marrow-derived cells of adult relative to young mice

**Figure 3.**
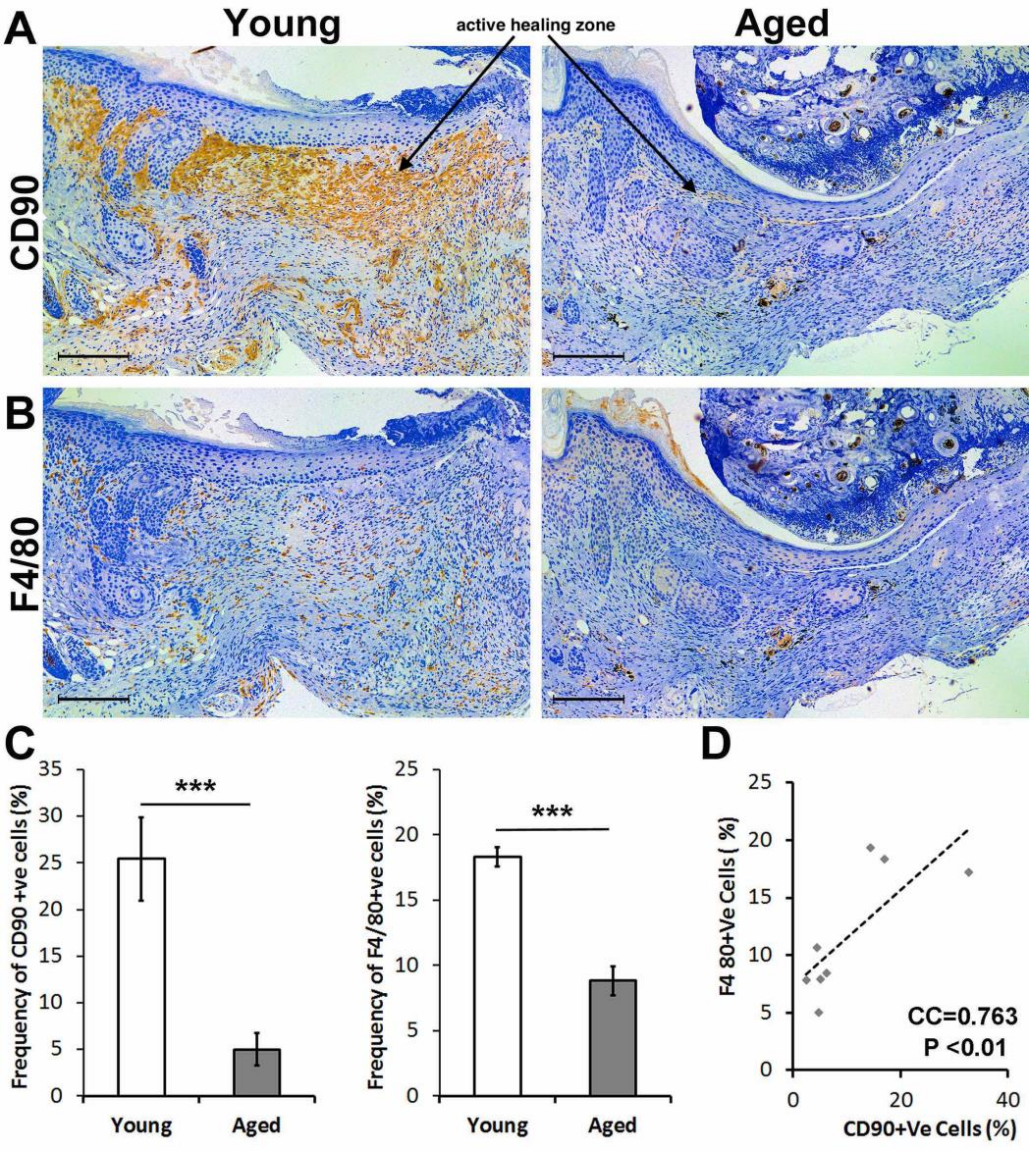
Adult mice have a decreased number of both F4/80+ macrophages, and CD90+ mesenchymal cells to the wound are compared to young mice. (A) Immunohistochemical staining of the healing skin two weeks post-injury shows a large population of CD90+ cells spanning from the junction of intact skin to the active healing zone (active healing zone marked with an arrow). This CD90+ cell population is significantly decreased in the adult mouse both at the junction and active healing area. (B) Immunohistochemical stain of F4/80 shows a decreased number of macrophages in the area of active healing in adult mice compared to young mice. (C) Quantification of the IHC stain shows significantly decreased frequency of CD90+ and F4/80+ cells in adult animals. (D) There is a significant correlation between frequency of CD90+ and F4/80+ cells in the wound sites of young animals (n=8, *P < 0.05, ***P <0.001).

Differentiated BM-MSCs were subjected to flow cytometry analysis and lineage differentiation experiments for further characterization. Although cells from both age groups were able to adhere to the plastic plate, form colonies, and differentiate towards mesoderm lineages, we observed a lower capacity in BM-MSCs derived from adult animals. MSCs populations from different age groups were characterized by flow cytometry to assess the percentage of cells positive for mesenchymal markers, CD73, CD105, CD90 and negative for hematopoietic markers (CD34, CD45). We observed statistically significant differences between the numbers of CD90+ mesenchymal stem cells of adult animals when compared to young animals (Fig. 3A, C). Immunohistochemical staining for CD90 indicated that many positive cells are present in the dermis in zones of active healing and the junction of intact skin in young mice as opposed to adult mice, which demonstrate sparse CD90+ cells (Fig. 3A, C).

**Figure 4.**
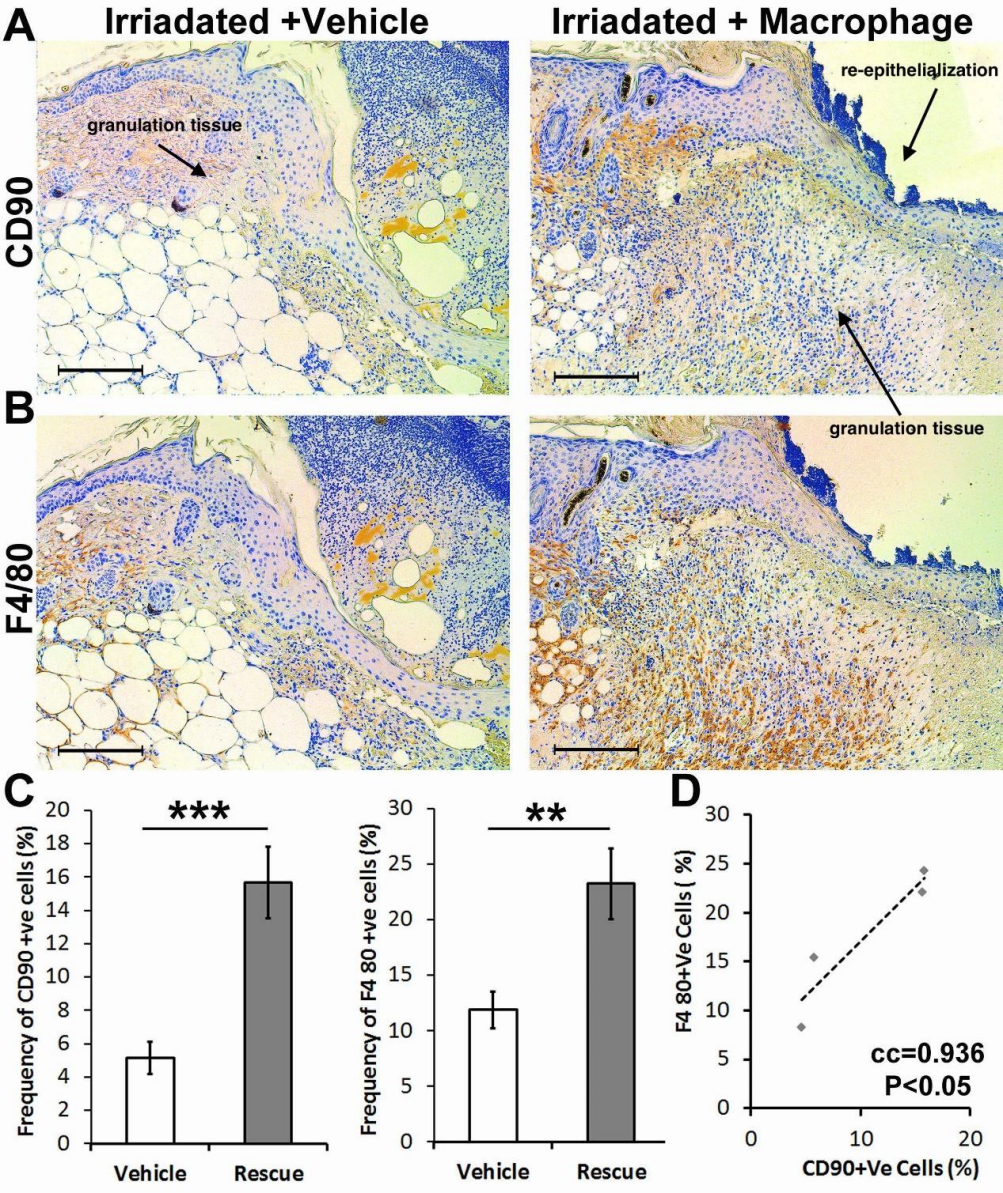
Topical addition of macrophages in irradiated young mice increases the number of CD90+ cells within the active wound area and rescues wound healing. (A,C) There is deficient wound healing in irradiated young mice with the vehicle, illustrated by the decreased amount of granulation tissue (labeled with an arrow). There is a decreased proportion of macrophages and CD90+ cells. (B,D) Applying macrophages, locally, increased granulation tissue formation and improved re-epithelialization (labeled with arrows), associated with a statistically significant increase in the proportion of macrophages and CD90+ cells. (C) There is a significant correlation between the number of macrophages and CD90+ cells in the active wound area (n=4, **P< 0.01, *** P<0.001, error bars are showing 95% confidence intervals).

We previously showed an essential role for macrophages during wound healing, in particular in the formation of granulation tissue and their interaction with mesenchymal-like cells [10]. Thus, we assessed if adult animals exhibit lower macrophage numbers versus young animals. Indeed, immunohistochemical staining indicated many F4/80+ macrophages in the dermis in zones of active healing and the junction of intact skin in young mice, similar to our observations with CD90 staining (Fig. 3B, D). Taken together, we demonstrated lower levels of CD90+ MSCs and macrophages in adult versus young mice, which could partly account for differential healing responses (e.g., poor granulation tissue formation, decreased extent of re-epithelialization) Therefore, we subsequently determined if there is a potential cell interplay between CD90+ MSCs and macrophages and if this interplay is a contributing factor to deficient healing in the elderly.

### Wound CD90+ cells are lower in adult animals and correlate with F4/80+ cell numbers

While Sca1+ cells in adult animals remain at the wound margin implying a possible migration deficiency in these cells, we observed a significant decrease in the number of CD90+ cells in the wound bed of adult animals (Fig. 3). Similarly, the number of F4/80+ myeloid cells were also lower in adult animals. While significant, this decrease is not as prominent as the decrease in CD90+ cells (5-fold decrease in the percentage of CD90+ cells vs. 2-fold decrease in the number of F4/80+ cells). Remarkably, the number of CD90+ cells and F4/80+ cells were positively correlated in the wound area (Fig. 3D). Although correlative, these findings suggest that these two cell types might synchronously migrate to the wound bed in young animals and that in adult animals, the myeloid cells continue to migrate to the wound center while CD90+ mesenchymal cells remain at the wound margin. We hypothesize that CD90 might be a surrogate marker for the physical interaction between mesenchymal stem cells and macrophages during skin healing.

### Macrophages steer CD90+ mesenchymal stem cells into the wound bed

To determine whether the association between CD90+ cells and macrophages in the wound bed is critical to migration, we examined healing in young mice depleted of bone marrow cells using a sub-lethal irradiation dose (2.6 Gy) 24 hours before wounding. As previously reported, the wounds in these irradiated mice were lacking macrophages and had a phenotype reminiscent of that seen in macrophage-deficient mice [10]. Simultaneously, these wounds were lacking CD90+ cells (Fig. 4). In an attempt to rescue this phenotype, macrophages harvested from the bone marrow of donor mice were applied to the wounds of irradiated mice. We observed that the deficient wound healing was partially rescued. In addition, the number of CD90+ cells was significantly increased in the wound bed of animals that received macrophages. Together, these findings suggest that macrophages steer CD90+ cells into the wound bed or provide a microenvironment that enhances the expression of CD90 in mesenchymal cells.

### Macrophages adhere to CD90+ mesenchymal stem cells

**Figure 5.**
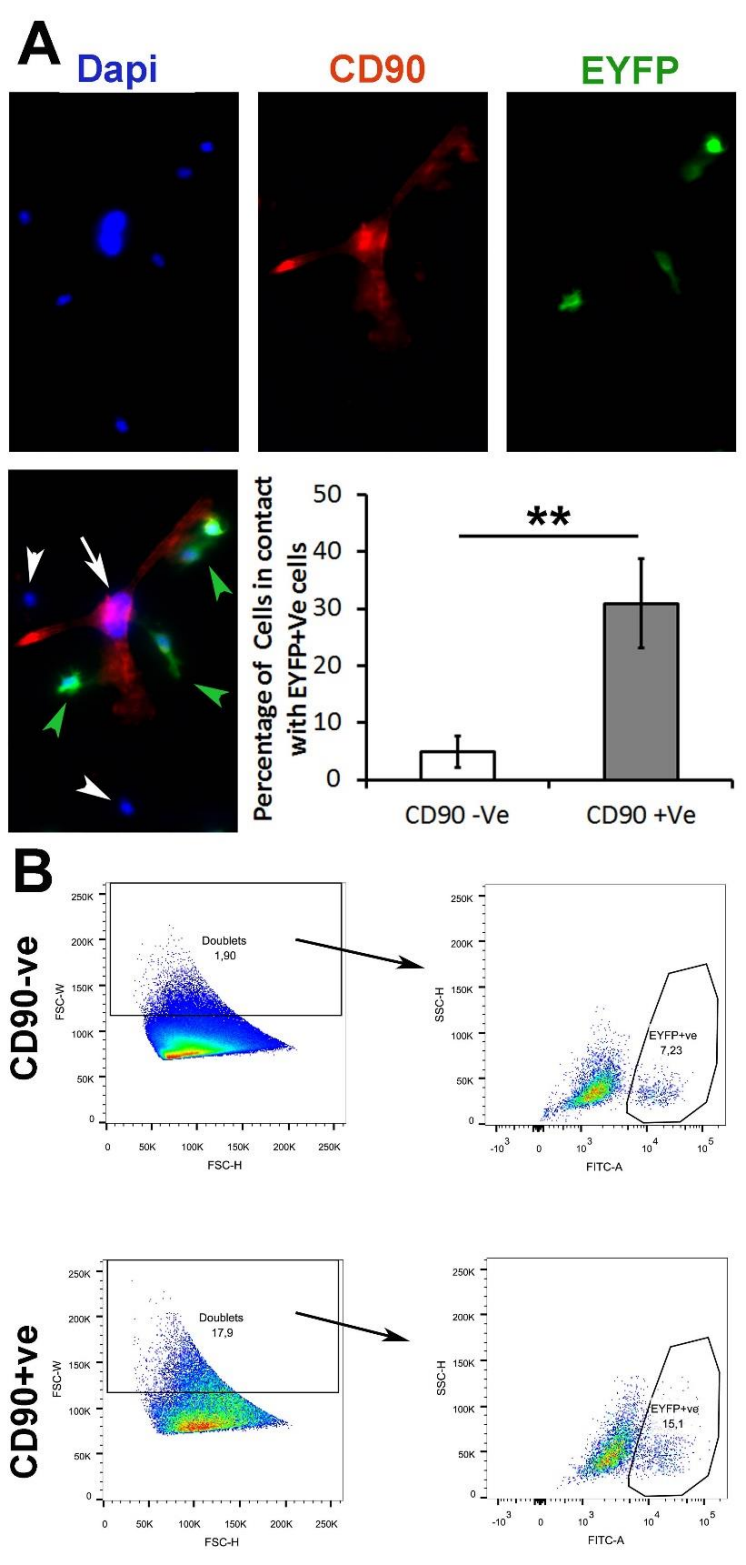
Macrophages may preferentially adhere to the CD90+ vs. CD90-ve MSCs. (A) Dapi (blue) and CD90 (red) immunofluorescence was performed after the addition of EYFP+ macrophages from young mice to a Boname marrow-derived MSCs. Macrophages preferentially adhere to CD90+ cells as compared to CD90-ve cells. (B) Macrophage (EYFP+ cells) and mesenchymal stem cell interaction were examined using flow cytometry staining for CD90. There is a significant increase in the percentage of doublet cells within the CD90+ cell population. There is also an increased proportion of EYFP+ cells within the CD90+ doublet cell population, suggesting a stronger interaction between CD90+ cells and macrophages than CD90- cells and macrophages (n=3 and ** P < 0.01, *** P <0.001, 95% confidence intervals).

In order to verify the nature of the interplay between CD90+ MSCs and macrophages, we subjected BM-MSCs of adult animals to the secretome of bone marrow-derived macrophages of young animals. We found that the secretome of macrophages did not significantly change the percentage of CD90+ cells *in vitro*. This suggests that the effects of macrophages on the migration of CD90+ cells to the wound bed are not mediated through paracrine effects. Next, we performed an adhesion assay where the macrophages obtained from young animals were added to the BM-MSCs derived from either young or adult animals. Interestingly, we noticed that more macrophages adhered to the MSCs of young animals (Supplementary Fig. 2) after washing and staining of the cells. To further verify the subpopulation of MSCs that are important for this adhesion, we performed another adhesion assay where the macrophages obtained from *Lysz-Cre ROSA-EYFP* young mouse bone marrow were seeded onto monolayers of cultured BM-MSCs obtained from *Gt(ROSA)26Sor^tm4(ACTB-tdTomato,-EGFP)Luo^*/J young mice. This mT/mG mouse expresses a red fluorescence when there is no Cre activity. While only 5% of CD90-ve MSCs had EYFP+ macrophages in their proximity, about 30% of CD90+ MSCs showed EYFP+ macrophages adhered to themselves (Fig. 5A and Supplementary Fig. 4).

### Macrophages, together with CD90+ mesenchymal stem cells, form doublets

While immunofluorescence staining revealed that macrophages have an affinity to adhere to the MSCs, prominently CD90+ MSCs, those experiments did not reveal the strength of this interaction. By performing the same adhesion assay using cells from young mice, we trypsinized the monolayer cells which have macrophages obtained from *Lysz-Cre ROSA-EYFP* on top (hence EYFP+ macrophages), washed and subjected them to flow cytometry analysis. Dissociated viable singlet or doublets events were gated by forward scatter-h, side scatter-w, and negative staining for propidium iodide^31^. Macrophage adherence to MSCs was gauged by the formation of doublets versus singlet events (single cells). Here, we demonstrated that almost 4 percent of total events were doublets. Interestingly, when gating for EYFP+ cells in both singlet and doublet populations, we demonstrated that doublets contained almost twice the number of macrophages (EYFP+ events). This suggests that macrophages seem to preferentially adhere to MSCs and form doublets rather than remain singlets (Supplementary Fig. 5).

To verify whether this higher affinity is within the CD90+ subpopulation, we stained the cell(s) with CD90 and gated out CD90+ cells from CD90-ve cells. Remarkably, the number of doublet events (macrophage adherence to MSCs) was 9-fold higher within the CD90+ events in comparison with CD90-ve events (Fig. 5). Within the CD90+ doublets, the number of EYFP+ macrophages was twice as the number of macrophages within CD90-ve events. Thus, these findings confirm our previous observation that macrophages preferentially adhere to to MSCs and have a higher affinity for CD90+ cells specifically.

## DISCUSSION

Impaired wound healing in elderly patients has been well documented and poses a substantial burden to health care economics as well as patient outcomes. In the context of burns, we recently showed that elderly patients have higher mortality, longer hospital stay, delayed inflammatory response and a delay wound healing predominantly due to the alteration in characteristics of progenitor cells [16]. Using adult animals and comparing them with their young counterparts, we uncover a mechanism that is essential for skin healing and that is impaired in adult animals. Adult animals have a smaller pool of MSCs, but these cells exhibit migration deficiency and instead remain restricted to the wound edge; this is more prominent in the CD90+ subpopulation. Importantly, our data shows that macrophages have greater affinity for CD90+ cells, illustrated by our flow cytometry data by the formation of doublets *in vitro*.

The interplay between mesenchymal cells and the myeloid lineage cells have been previously demonstrated during healing and cancer propagations [10, 32-35]. However, we uncover here that the CD90+ subpopulation of the MSCs may preferentially adhere to macrophages, although our findings are correlative. In addition, our skin healing experiments showed that aged animals fail to show granulation tissue formation at the center of burn injury (~2.5 cm width). While the deficient healing in aged animals may be associated with several deficient factors during the process of aging [36], we found that the limited pool of Sca1+ cells in adult animals remain stuck at the injury junction and are unable to contribute to skin healing. In an *in vitro* experiment, the adult bone marrow-derived MSCs showed a deficient migratory phenotype in comparison with the MSCs from young animals, both functionally in the scratch assay and gene expression assay. This shows that MSCs from adult animals are intrinsically deficient in migration. Cell adhesion and cell migration are tied to each other, and one affects the other one. In particular, forming an adhesion complex or disassembly of adhesion drives cell migration [37-39]. This prompted us study the relationship between MSC migration to adhesion. In particular, we hypothesized that the deficient migration of MSCs in adult animals may be partly due to their deficiency to adhere to macrophages. We observed that MSCs of adult animals have a lower proportion of CD90+ subpopulation. With this, we compared the adhesion phenotype of CD90+ MSCs versus CD90-ve MSCs. In cancer, it has been shown that tumor-associated monocytes and macrophages provide a niche that upregulates the expression of CD90 and that this partly mediates the physical interaction of cancer stem cells with myeloid cells [40].

We observed a significant decrease in the number of CD90+ cells and F4/80+ macrophages in the wound bed of adult animals. However, the decrease in the proportion of F4/80+ cells is modest in comparison with the significant drop in the proportion of CD90+ cells. This suggests that a cell-cell interaction while migrating to the wound bed. Indeed, in adult animals, these myeloid cells continue to migrate to the wound center while CD90+ mesenchymal cells remain at the corner of the wound. Subsequently, we used irradiation in young animals to study whether CD90 might be a surrogate marker that predicts MSCs-macrophage adhesion during skin healing. Irradiation ablates macrophages and alters skin healing, and we used this model to further explore this phenomenon as a proof of concept. Although we did not compare age-related changes in healing with macrophage treatment, we recapitulate the features seen in aged/adult murine healing (i.e., lower macrophage levels). Subsequent studies in aged/adult mice treated with macrophages from young donors would be of interest.

We showed here that local application of macrophages not only increased the number of macrophages in the wound bed but also increased the pool of CD90+ cells, suggesting that applied macrophages steer CD90+cells from the host animal into the wound bed. It is also possible that the applied macrophages provide a microenvironment that enhances the expression of CD90 in mesenchymal cells. Given that the secretome of bone marrow-derived macrophages did not significantly change the percentage of CD90+cells *in vitro*, we focused on steering as a mechanism of action and asked whether this was due to the indirect effect of macrophages where macrophage secretome can indirectly affect MSCs migration or the macrophage can directly adhere to the CD90+ MSCs and migrate to the wound bed. In an *in vitro* adhesion assay, we showed that after adding macrophages on top of a monolayer culture of BM-MSCs, macrophages preferentially adhere to CD90+ cells. The interplay of the stem cells and their niche cells has been studied where the cells form doublets in flow cytometry [31, 41]. Performing the very same adhesion assay and trypsinizing cells, we observed a dramatic increase in the proportion of doublets within CD90+ events. This suggests that CD90+ cells preferentially form doublets with other cells. Interestingly, the proportion of EYFP+ macrophages is higher within the doublets of CD90+ cells, suggesting that a substantial percentage of the cells that adhered to the CD90+ cells are macrophages.

There are several limitations and avenues for future directions in our study. Firstly, while we elaborate on the significance of the CD90+ MSC and macrophage interaction, we have not applied these cells in isolation on the wound and compared healing. However, previous work demonstrated the importance of F4/80+ macrophage infiltration in murine wound healing after burn, illustrating how decreased macrophage levels and altered polarization state impairs healing [42]. Furthermore, human studies have indicated that local application of macrophages is beneficial in other models of poor healing such as chronic ulcers [43]. Similarly, there is prior data demonstrating the beneficial effect of MSC application in wound healing (e.g., cell proliferation, angiogenesis, collagen synthesis) [44,45]. Additionally, while our data demonstrates that macrophages and CD90+ cells may putatively adhere to each other and likely migrate together to the wound bed, it remains unknown whether this affinity anchors a functional junction (e.g., desmosome, hemidesmosome). Further electron microscopy may provide insight into the nature of this adhesion, which is out of the scope of this report at the moment. Additionally, another limitation is that we focused on the correlation between CD90+ MSCs and macrophages. However, there are putatively other cell types correlated with CD90+ MSCs such as endothelial cells. Additional studies investigating cell-cell interactions would be of utility in order to identify if these interactions are integral to the wound healing process or if CD90+ MSC/macrophage is a novel, unique relationship.

## Conclusions

Macrophages correlated with and may preferentially adhere to the CD90+ MSCs, enabling these cells to migrate together to the wound bed. This adhesion is likely impaired in adult animals, with MSCs remaining at the border of the wound while macrophages migrating to the center of the wound. This contributes to clinically significant deficient skin healing in adult animals. This study introduces a potential interplay between mesenchymal stem cells and myeloid lineage cells exists, and our findings provide more insight into this interaction during skin healing.

## Supplementary Materials

The Supplementary data can be found online at: www.aginganddisease.org/EN/10.14336/AD.2021.1008.


