# Supplementary material for: Aging Impairs the Cellular Interplay between Myeloid Cells and Mesenchymal Cells during Skin Healing in Mice
Source: Aging Dis. 2022 Apr 1;13(2):540–51. doi: 10.14336/AD.2021.1008 (PMC8947831; doi:10.14336/AD.2021.1008)
Supplement: Supplementary file 1 [file AD-13-2-540-s.pdf]

SUPPLEMENTARY DATA

Aging Impairs the Cellular Interplay between Myeloid Cells and Mesenchymal Cells during Skin Healing in Mice

Saeid Amini-Nik[2,3,4,6,#,*], Abdikarim Abdullahi[1,2,#], Roohi Vinaik[1,2], Ren Jie Robert Yao[1,2], Nancy Yu[2], Andrea Datu[2], Cassandra Belo[2], Marc G Jeschke[1,2,4,5,6*]

© 2021. Amini-Nik S et al. Published online at http://www.aginganddisease.org/EN/10.14336/AD.2021.1008

SUPPLEMENTARY DATA

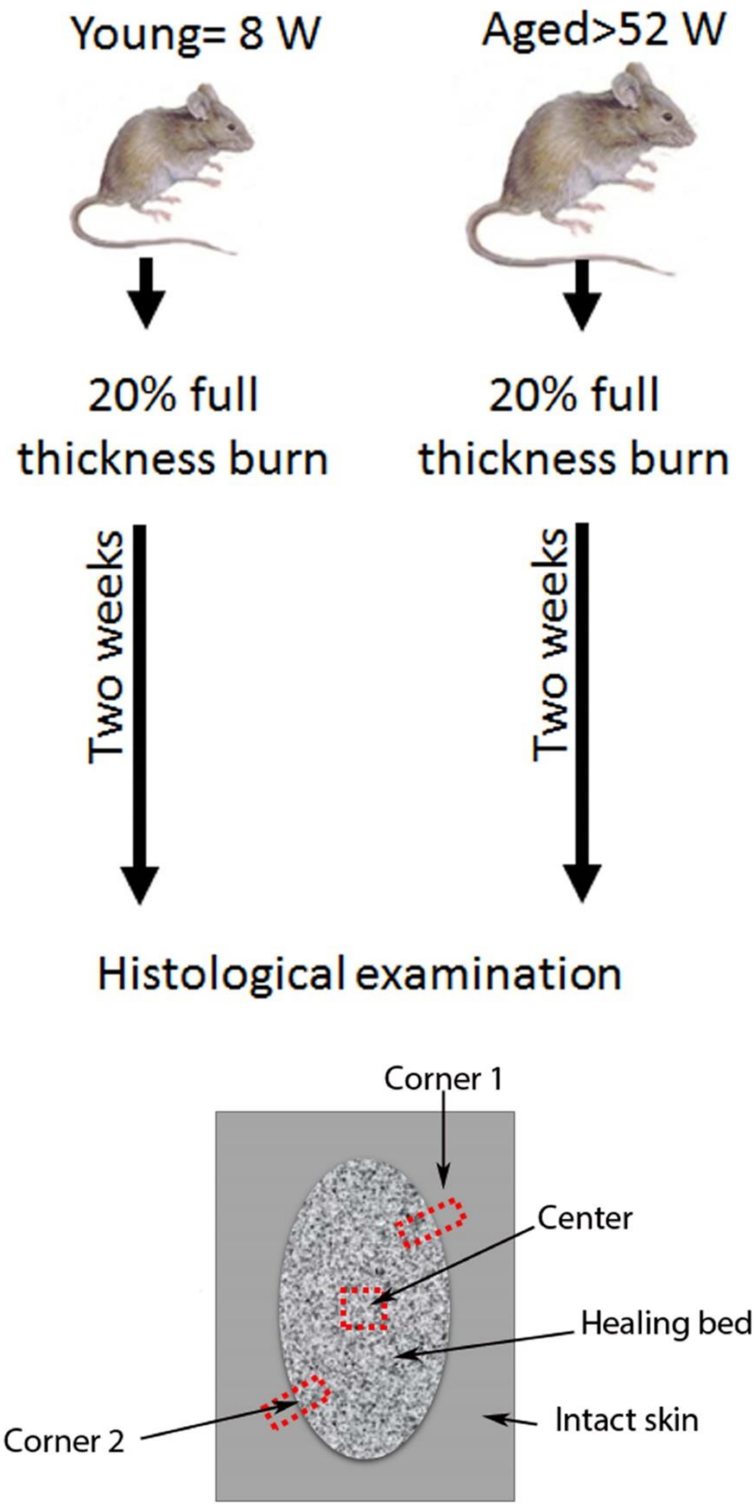

Supplementary Figure 1. Thermal injury model on dorsum skin of mice and isolation of healing tissue for histological and other proteomics and transcriptomics approaches.

© 2021. Amini-Nik S et al. Published online at http://www.aginganddisease.org/EN/10.14336/AD.2021.1008

SUPPLEMENTARY DATA

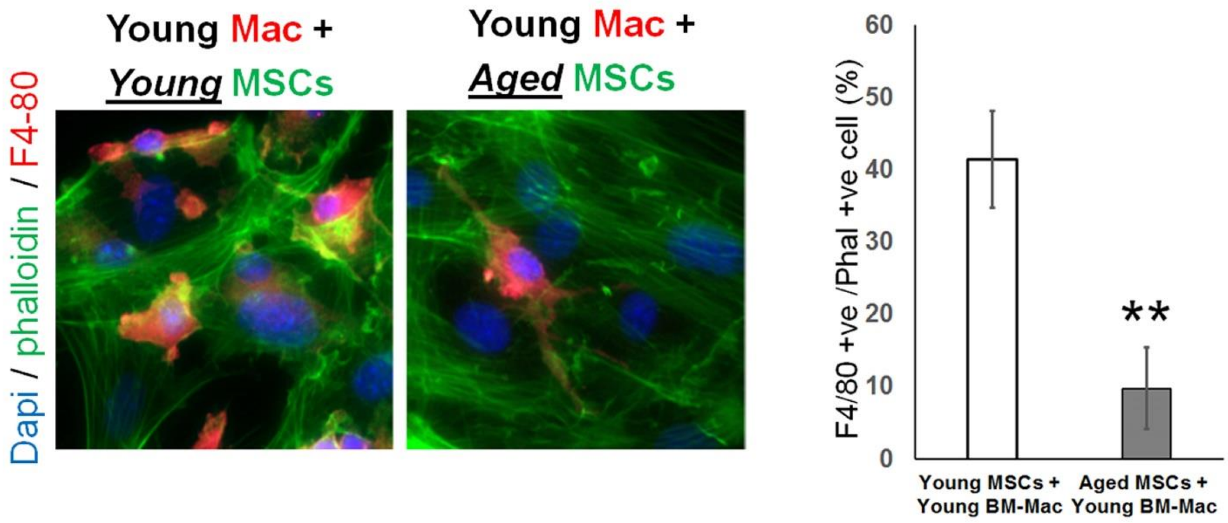

Supplementary Figure 2. Macrophages adhere to the adult MSCs in a lesser extent. Fewer EYFP+ macrophages attached to the adult MSCs as compared to young MSCs in adhesion experiments (** $P < 0.01$, error bars are showing 95% confidence intervals).

© 2021. Amini-Nik S et al. Published online at http://www.aginganddisease.org/EN/10.14336/AD.2021.1008

SUPPLEMENTARY DATA

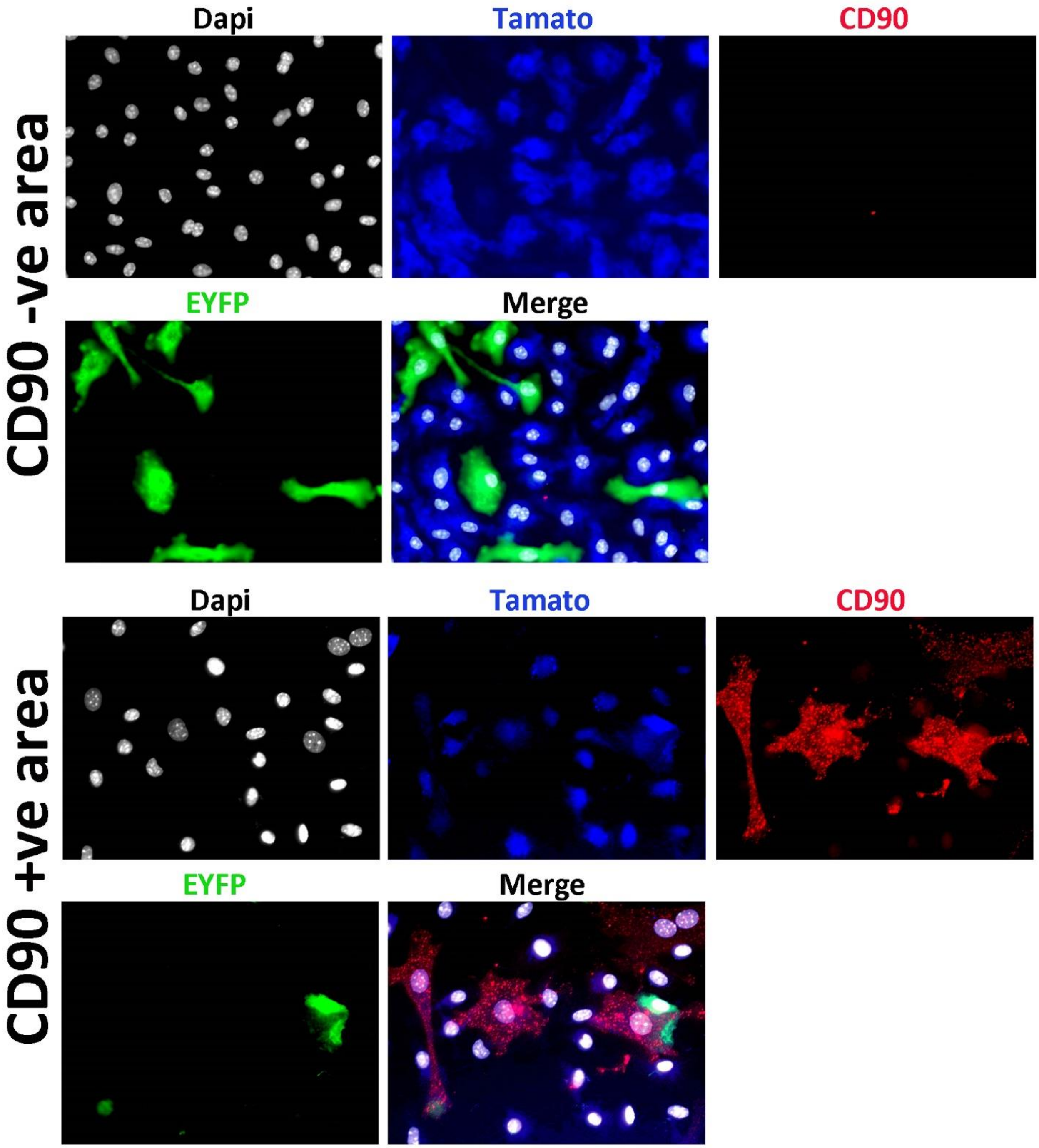

Supplementary Figure 3. Macrophages from young mice preferentially adhere to CD90+ mesenchymal cells.

© 2021. Amini-Nik S et al. Published online at http://www.aginganddisease.org/EN/10.14336/AD.2021.1008

SUPPLEMENTARY DATA

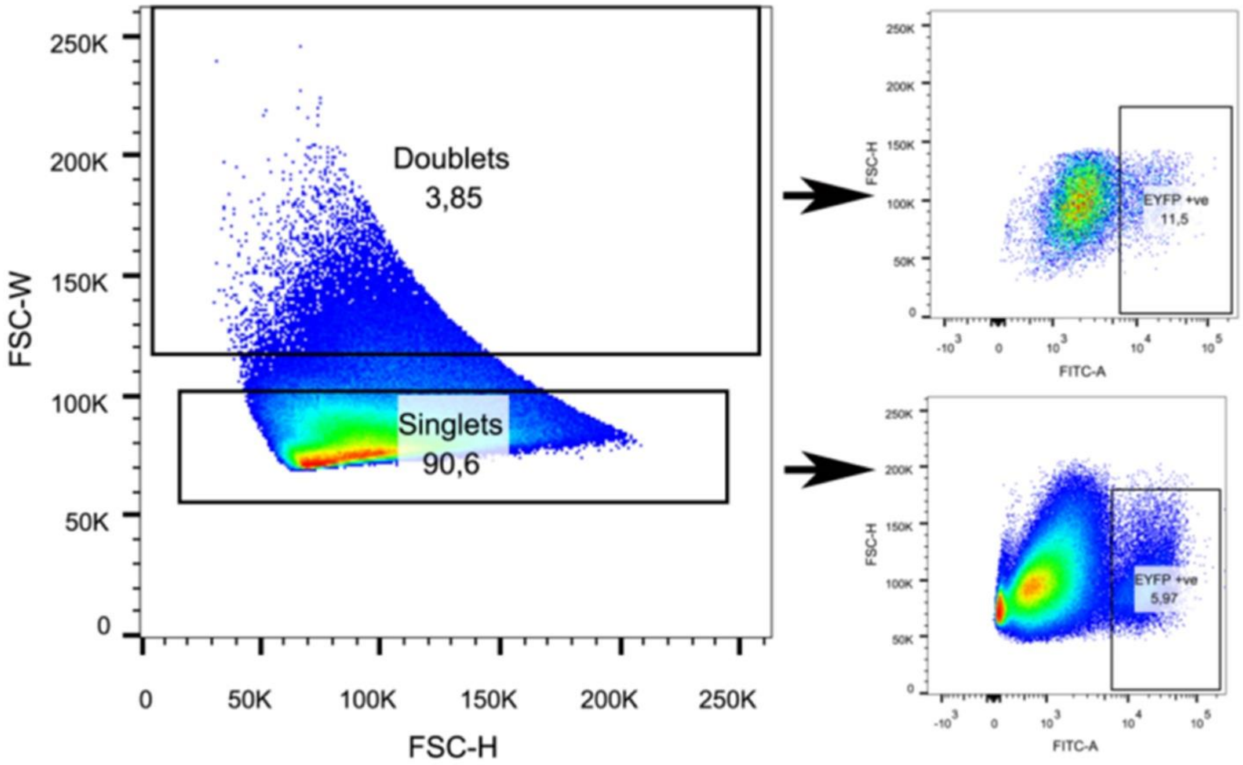

Supplementary Figure 4. Macrophages, together with CD90+ mesenchymal stem cells, make doublets. Flow cytometry of trypsinized cells obtained from young mice after adhesion of EYFP+ macrophages is showing that a substantial population of the dissociated viable events are doublets events and the number of EYFP+ macrophages are significantly more in doublet population in compare with singlet population.

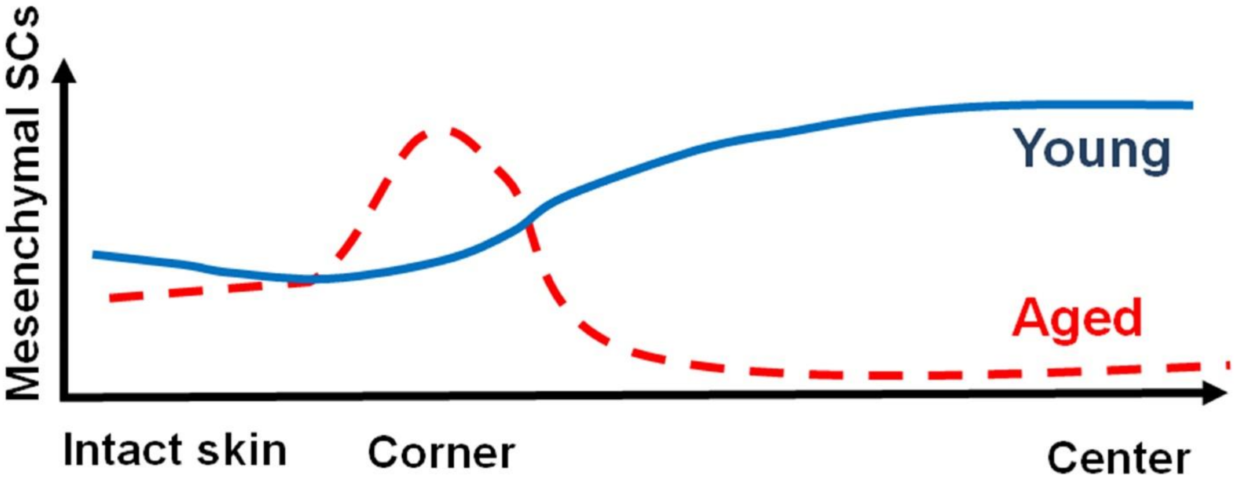

Supplementary Figure 5. Schematic illustration of the spatial distribution of MSCs in young vs. adult animals.

© 2021. Amini-Nik S et al. Published online at http://www.aginganddisease.org/EN/10.14336/AD.2021.1008

Supplementary Table 1. RNA analysis shows the down-regulation of key adhesion genes in CD90-ve MSCs as compared to CD90+ MSCs.

Symbol	Description	Fold Regulation
Dpp4	Dipeptidylpeptidase 4	-11.9211
Egf	Epidermal growth factor	-6.1064
Actn3	Actinin alpha 3	-5.1145
Rho	Rhodopsin	-4.1576
Cav1	Caveolin 1, caveolae protein	-2.9902
Wasf1	WASP family 1	-2.6788
Fap	Fibroblast activation protein	-2.5253
Pld1	Phospholipase D1	-2.3191
Pxn	Paxillin	-2.0829
Ptpn1	Protein tyrosine phosphatase, non-receptor type 1	-2.0278

FACS sorted CD90+ and CD90-ve from bone marrow-derived MSCs of young mice show a decreased expression of adhesion-related genes in CD90-ve MSCs.

© 2021. Amini-Nik S et al. Published online at http://www.aginganddisease.org/EN/10.14336/AD.2021.1008
